# Cost-Effectiveness of Physical Activity among Women with Menopause Symptoms: Findings from a Randomised Controlled Trial

**DOI:** 10.1371/journal.pone.0135099

**Published:** 2015-08-10

**Authors:** Päivi Kolu, Jani Raitanen, Clas-Håkan Nygård, Eija Tomás, Riitta Luoto

**Affiliations:** 1 UKK Institute for Health Promotion; Tampere, Finland; 2 School of Health Sciences, University of Tampere; Tampere, Finland; 3 Tampere University Hospital; Tampere, Finland; Deakin University, AUSTRALIA

## Abstract

Menopause is a period that may predispose one to a decrease in muscle strength, cardiorespiratory fitness, and quality of life. A study was carried out to evaluate the cost-effectiveness of physical activity among women displaying symptoms of menopause. The cost-effectiveness analysis was based on data from a six-month randomised controlled trial (*n* = 151). The women in the intervention group engaged in an unsupervised session of at least 50 minutes of physical activity four times a week. The control group continued their physical activity as before. An incremental cost-effectiveness ratio (ICER) was calculated in terms of maximal oxygen consumption, lean muscle mass, and quality-adjusted life years (QALYs) gained. A bootstrap technique was utilised to estimate uncertainty around the point estimate for ICER associated with the intervention. The mean total cost in the intervention group was €1,307 (SEM: €311) and in the control group was €1,253 (SEM: €279, *p* = 0.10) per person. The mean intervention cost was €208 per person. After six months of the behaviour-change intervention, the ICER was €63 for a 1 ml/kg/min improvement in cardiorespiratory fitness, the additional cost per one-gram increase in lean muscle mass was €126, and the cost per QALY gained was €46. According to the findings, physical activity among menopausal women was cost-effective for cardiorespiratory fitness, for lean muscle mass, and for QALYs gained, since the intervention was more effective than the actions within the control group and the additional effects of physical activity were gained at a very low price. From the societal perspective, the intervention used may promote ability to work and thereby save on further costs associated with early retirement or disability pension if the physical-activity level remains at least the same as during the intervention.

## Introduction

Menopause, the time in a woman’s life that leads to the end of fertility [1], occurs internationally at, on average, age 45 to 52 [2]. During menopause, the decreasing amount of various hormones and steroids, such as oestrogen and progesterone, may cause depression and anxiety but also vasomotor symptoms, including hot flushes [1, 3]. In addition, hormonal reduction speeds up ageing while also increasing the risk of chronic diseases such as osteoporosis and cardiovascular disease, on account of elevated triglycerides, LDL cholesterol, and total cholesterol [1, 3]. Menopause is also a period that may predispose one to a decrease in health-related quality of life (HRQoL) and muscle strength while increasing body mass index (BMI) [1, 3].

According to global physical-activity recommendations, adults should engage in, at minimum, 150 minutes of at least moderate-intensity physical activity a week, with the suggested approach being sessions of 10 minutes duration or more, at least three times per week [4]. Though a physically active lifestyle has been shown to have an important role in health, 26% of women at menopausal age (45 to 64 years) did not meet the physical-activity recommendation for cardiorespiratory fitness in Finland, on average [5]. Internationally the prevalence of physical inactivity was even greater, since in Americas and the eastern Mediterranean region around 45% of women of menopausal age (45 to 59 years), and in Europe overall 37%, did not meet the physical-activity recommendation [6].

Physical activity of at least moderate intensity has been shown to prevent cardiovascular disease among menopausal women because of improved blood lipids and may also decrease body fat, thereby reducing a risk factor for diabetes [7]. Moreover, physical activity may reduce menopause symptoms such as hot flushes and thereby improve quality of life [8, 9]. Even though menopause is a phenomenon that concerns all women to some extent, no cost-effectiveness studies related to physical activity and menopausal women without hormone therapy had been done prior to the work described here. The topic is important from a societal perspective because regular physical activity of at least moderate intensity may help women of menopausal age to maintain their capacity for work, via improved cardiorespiratory fitness and muscle strength, both of which gradually decrease after age 40–50 [10]. It is of societal relevance that decreased capacity for work may leave one prone to early retirement and to disability pension. In addition, physical activity may help to keep health-care costs under control by preventing symptoms and disorders related to menopause.

We have earlier published the main results from our randomised trial (RCT) among menopausal women. The aim of that RCT was to estimate whether aerobic training had an effect on frequency of hot flushes or quality of life [9]. Results of the study showed that HRQoL was improved among the intervention group with respect to physical functioning (*p* = 0.049) and physical role limitation (*p* = 0.017). Cardiorespiratory fitness improved in the intervention group from 31.7 to 32.6 ml/kg/min and decreased in the control group from 31.5 to 31.4 ml/kg/min (*p* = 0.008), and lean muscle mass rose significantly in the intervention group relative to controls (0.57 kg in contrast to 0.15 kg, *p* = 0.046) [8]. The aim of the study reported upon here was to assess the cost-effectiveness of at least moderate-intensity physical activity for cardiorespiratory fitness, for lean muscle mass, and per quality-adjusted life year (QALY) gained among menopausal women.

## Material and Methods

### Study design and participants

The cost-effectiveness analysis was based on a randomised controlled trial (*n* = 151), the main objective of which was to evaluate the effect of unsupervised physical activity on hot flushes and quality of life among menopausal women (trial registration ISRCTN54690027; see http://www.controlled-trials.com/) [9, 11]. Participants were enrolled via newspaper advertisements between January and March 2009 in Finland. The duration of the cost-effectiveness trial was six months, so it represented only a half-year follow-up. The study was approved by the medical ethics committees of the Pirkanmaa hospital district, and participants’ informed consent was obtained with a signature at the first appointment. The research was conducted in accordance with prevailing ethics principles. Collection of data for the economic evaluation was performed during the original RCT study.

The inclusion criteria were daily hot flushes, an age of 40–63, neither current hormone therapy nor use of it within the previous three months, a sedentary lifestyle (physical activity no more than twice a week), and 6–36 months since last menses. Women who were physically active (defined as engaging in at least 30 minutes of vigorous physical activity at least three times a week), were obese (BMI > 35 kg/m²), or had a physical condition preventing physical activity (cardiorespiratory or orthopaedic disease or a medication influencing heart rate, such as beta-blockers) were excluded.

### Outcome measurements

The cost-effectiveness of the intervention was evaluated in terms of cardiorespiratory fitness (VO_2_Max), lean muscle mass (kg), and QALYs gained. Cardiorespiratory fitness was evaluated by means of the UKK two-kilometre walk test [12] at the beginning and end of the trial. In its consideration of lean muscle mass, the cost-effectiveness analysis measured it via dual x-ray absorptiometry (DXA) at the start and the end of the trial. The QALY data were calculated from the SF-6D score derived from the original SF-36 data, which is a validated instrument for measuring the physical and mental components of quality of life [13–15]. The index score covers eight dimensions of HRQoL: physical functioning, physical role limitation, bodily pain, general health, vitality, social functioning, emotional role limitation, and mental health. The index score is expressed by a single number, from 0 (representing the death) to 1 (denoting the best health state). To calculate QALYs gained, after the difference in index score between six months and the baseline was taken into account, the index was multiplied by the expected lifetime calculated for each woman separately. The weighting of the index score was based on the UK population [13]. The HRQoL data were collected via questionnaires completed at the beginning and at the end of the six-month trial. Even though the frequency of hot flushes and responses to a women’s health questionnaire (WHQ) were used as outcomes in the original study, they were not employed as outcomes in the cost-effectiveness analysis, because the results could not be presented by means of one index number.

### The intervention group

The participants in the training group (*n* = 74) were instructed to engage in four unsupervised sessions of physical activity per week, of at least 50 minutes’ duration each. At least two physical-activity sessions were to consist of walking or Nordic walking, which is walking with poles that are intended to increase the efficiency of the exercise. Walking was chosen as a mode of physical activity because of earlier demonstration of favourable effects of walking on menopausal women’s body composition and cardiorespiratory fitness [7]. To achieve at least moderate-intensity physical activity, the women were instructed to exercise at a subjective level of ‘somewhat hard’ (corresponding to 13–16 on a 6–20 scale) [16], which is consistent with the intensity of aerobic training recommended in physical-activity guidelines [4]. The required subjective intensity corresponded to about 64–80% of maximal heart rate [16].

For obtaining objective data on intensity of physical activity, the intervention group wore heart-rate monitor belts by Suunto during the training sessions. These collected data on both the intensity and the duration of the sessions. To ensure collection of data on physical activity, the heart-rate monitor belts were complemented with gathering of information via a questionnaire on physical activity during the intervention. The physical-activity questionnaire included items on the type of physical activity, minutes of activity, and its intensity. For sustained commitment to the trial, the members of the intervention group received feedback on comparison to recommendations from an aerobic training instructor, every second week. The personal feedback given every other week concentrated on intensity and amount of training in comparison to the recommendations but also on motivation to continue the physical activity in spite of physical discomfort or symptoms of menopause, which are commonplace among physically inactive women [1]. The aerobic training instructor took advantage of information from the heart-rate belts in order to increase the effectiveness of participants’ physical activity progressively from 64% (at the beginning of the intervention) to 80% of maximal heart rate (at the end of the intervention).

In addition, the women in the intervention group were offered an opportunity to take part in instructor-led aerobics sessions twice a week. Participants in both groups were given personal mobile phones to report information on the intensity of their hot flushes, which was the main outcome in the original intervention. Moreover, the intervention and control group both took part in, on average, twice-monthly lectures that covered topics related to physical activity and women’s health. In the course of the six months, there were 14 dropouts (representing 9.3% of the sample) from the intervention group.

### The control group

The women in the control group (*n* = 77) were advised to continue their physical activity as before. However, these participants were encouraged to take part in the same twice-monthly lectures as those in the intervention group. To control for maintenance of physical activity, a questionnaire was used to ask women in the control group about their physical-activity habits over the past three months at both the beginning and the end of the intervention. The number of persons who dropped out from the control group was eight (5.3% of the total). In addition, three persons’ data were excluded from analysis for reason of missing data.

### Economic evaluation

The economic evaluation included intervention costs, health-care costs for the municipality, costs borne by the patient, and productivity costs from the societal perspective. The travel expenses and time costs related to the use of health services should have featured in the calculation, but this information was not available. The opportunity cost, which in economics denotes the value of the best alternative forgone, was not estimated, because there was no precise information available on the time spent engaging in physical activity during the intervention period by the control group.

The information on the use of health-care services (visits to physicians and to nurses, including occupational health nurses), inpatient days, and medication was obtained via questionnaires at the beginning and at the end of the trial. Medications received on inpatient days were not included in the ‘medication costs’ category, because they were already covered by daily inpatient costs [17]. The medication cost includes both the patient- and the society-borne amount; the 2009 medication costs were 42% covered by the patient, with society contributing the rest of the amount [18]. Health-care costs were calculated from the average national unit costs for health care [17]. The unit costs of visits to physicians and to nurses included salary costs and administrative costs but not laboratory expenses. In addition, the unit cost of inpatient hospital days included the daily inpatient-care charge [17]. Costs were calculated for the full six-month physical-activity trial period. Unit costs, productivity costs, and medication costs were entered at 2009 price levels in euros.

The intervention costs were obtained directly from the trial and included the cost of the work contribution of the aerobic training instructor and the physicians’ contribution. The work contribution of the aerobic training instructor consisted of leading one-hour aerobic training sessions twice a week and giving personal feedback every second week on the heart-rate belt data with regard to realisation of physical activity. The cost of the mobile phones and of the heart-rate belts were also included in the intervention costs. The physicians’ work contribution consisted of implementation of lectures related to women’s health, and salary expenses.

Lost productivity was evaluated by means of self-reported information on absence from part- or full-time work via a questionnaire at the end of the trial. Salary costs were calculated from women’s average national monthly salary scales in 2009 [19], multiplied by 1.3 so as to encompass related expenses. The cost calculation assumed 220 workdays a year.

### Statistical analysis

Baseline characteristics, costs, and effects were reported as means and standard error of mean (SEM) values or as frequencies and percentages. The association between the groups was tested with the Mann-Whitney U test. In the case of some data related to results for cardiorespiratory fitness, lean muscle mass, and the SF-36 questionnaire, imputations for missing values were performed via multiple imputation technique via the Stata software (with the mi command). Cost data were available for all participants. For evaluation of cost-effectiveness, the differences between groups were analysed via a non-parametric bootstrap approach. Cost-effectiveness was expressed in terms of incremental cost-effectiveness ratios (ICERs), which indicate the amount of money that is required to increase cardiorespiratory fitness, lean muscle mass, and QALYs gained. Health-related quality of life was judged on the basis of the standardised SF-36 questionnaire, which refers to 36 physical and mental components of everyday life during the past four weeks [13–15]. A bootstrap technique with 5,000 replications [20] and cost-effectiveness acceptability curves were used to assess 95% confidence intervals for purposes of analysing the uncertainty around the point estimate of the ICER. The formula for calculation of ICER is

$$ICER\;=\;{\left(Cost_{Intervention}-Cost_{Control}\right)}_{}/\;(Effect_{Intervention}{-}_{}Effect_{Control)}.$$

The confidence intervals (CIs) were calculated by means of a bias-corrected and accelerated (BCa) method proposed by Efron [21]. The BCa interval is given in terms of percentiles of the bootstrap distribution of differences in means, but the percentiles used are chosen after correction for skewness or ‘acceleration’ â and bias [22]. The results were considered to be statistically significant if *p* < 0.05. Analyses were performed with SPSS (version 22) and Stata (version 12.1) statistics software.

### Sensitivity analysis

To evaluate the robustness of the findings, we performed sensitivity analysis in three separate ways. Firstly, aerobic training instructors’ costs as a component of the intervention costs were doubled. The reason for doubling the value of aerobic training instructor costs per person was the brevity of the counselling sessions realised: there was only 16 minutes per person, every second week. For women with menopause symptoms, twice the amount of time (32 minutes) was more realistic. Secondly, we carried out sensitivity analysis with equal mobile-phone and heart-monitor costs across the two groups. For greater reliability of the results, all measurements performed for the intervention and control group should have been the same between the groups. The third sensitivity analysis was a complete case analysis. This entailed including only those participants whose data were available in full, without imputation, with respect to cardiorespiratory fitness, lean muscle mass, and the SF-36 questionnaire.

## Results

Of the 151 women with menopause symptoms, 74 (49%) were in the intervention group and 77 (51%) were in the control group (see Table 1).

**Table 1:** Baseline characteristics (mean and standard error of mean or frequency and proportion).

	Intervention group (*n* = 74)	Control group (*n* = 77)
Age	54.5 (0.44)	54.2 (0.43)
Weight (kg)	70.5 (1.32)	71.7 (1.43)
BMI (kg/m^2^)	26.3 (0.46)	26.9 (0.49)
University degree (%)	18 (24.3)	20 (26.0)
Employment (%)	61 (83.6)	59 (77.6)
Regular or occasional smoker (%)	13 (18.1)	9 (11.7)
Maximal oxygen consumption (ml/kg/min)	31.7 (0.63)	31.5 (0.54)
Lean muscle mass	40.1 (0.51)	40.2 (0.55)
Use of health-care services in the previous 12 months		
Visits to a physician	3.51 (0.42)	3.05 (0.31)
Visits to a nurse (incl. occupational health nurses)	1.04 (0.18)	1.11 (0.15)
Inpatient days	1.23 (0.83)	0.35 (0.12)
Health-related quality of life		
SF-36 index score (between 0 and 1)	0.734 (0.0144)	0.730 (0.0121)

### Costs

The per-person mean total cost was nearly the same between the intervention and the control group (at €1,307 and €1,253, respectively; *p* = 0.10); see Table 2. The direct costs and productivity costs were nine per cent lower in the intervention group than for the control group (€1,100 and €1,208, respectively; *p* = 0.53). The mean cost per person during the intervention was €208, which was 16% of the total costs for the intervention group. There were no statistically significant differences between the intervention and control group in direct costs (visits to a physician or to a nurse, medication, and days of inpatient care) or absence from work (see the costs detailed in Table 2). The largest proportion of the intervention costs consisted of the aerobic-training work contribution, which came to 3.5 hours per participant over the six months (see Table 2).

**Table 2:** Direct costs, productivity costs, and costs of intervention.

		Intervention group (*n* = 74)		Control group (*n* = 77)		
	Unit cost^a^ (EUR)	Number of units	Mean cost^a^ (EUR)	Number of units	Mean cost^a^ (EUR)	*p*-value^b^
**Direct costs**						
Visits to a physician^c^	104/visit	1.53	159 (20.5)	1.96	203 (26.0)	0.40^c^
Visits to a nurse (incl. occupational health nurses)^c^	76/visit	0.44	33 (6.3)	0.44	34 (7.2)	0.75
Medication			48 (31.0)		31 (17.2)	0.56
Inpatient days	785/day	0.02	12 (11.9)	0.10	76 (35.3)	0.12
**Productivity costs**						
Absence from work (€3,470/month)	189/day	4.70	848 (327)	4.84	864 (268)	0.34
**Total costs**			**1,100 (311)**		**1,208 (279)**	0.53
**Costs of the intervention**						
Lectures for both groups	70/hour	11.25 hours	5	11.25 hours	5	
Mobile phones (incl. prepaid card)			40		40	
Heart-rate monitors			50			
Aerobic lessons	27/hour	52 hours	19		-	
Supplemental aerobic training instructor’s work contribution per person	27/hour	3.5 hours	94		-	
**Total costs**			**1,307 (311)**		**1,253 (279)**	0.10

^a^ Costs rounded to the nearest euro.

^b^ Mann-Whitney U test.

^c^ Including outpatient-care charge.

### Cost-effectiveness

**Fig 1:**
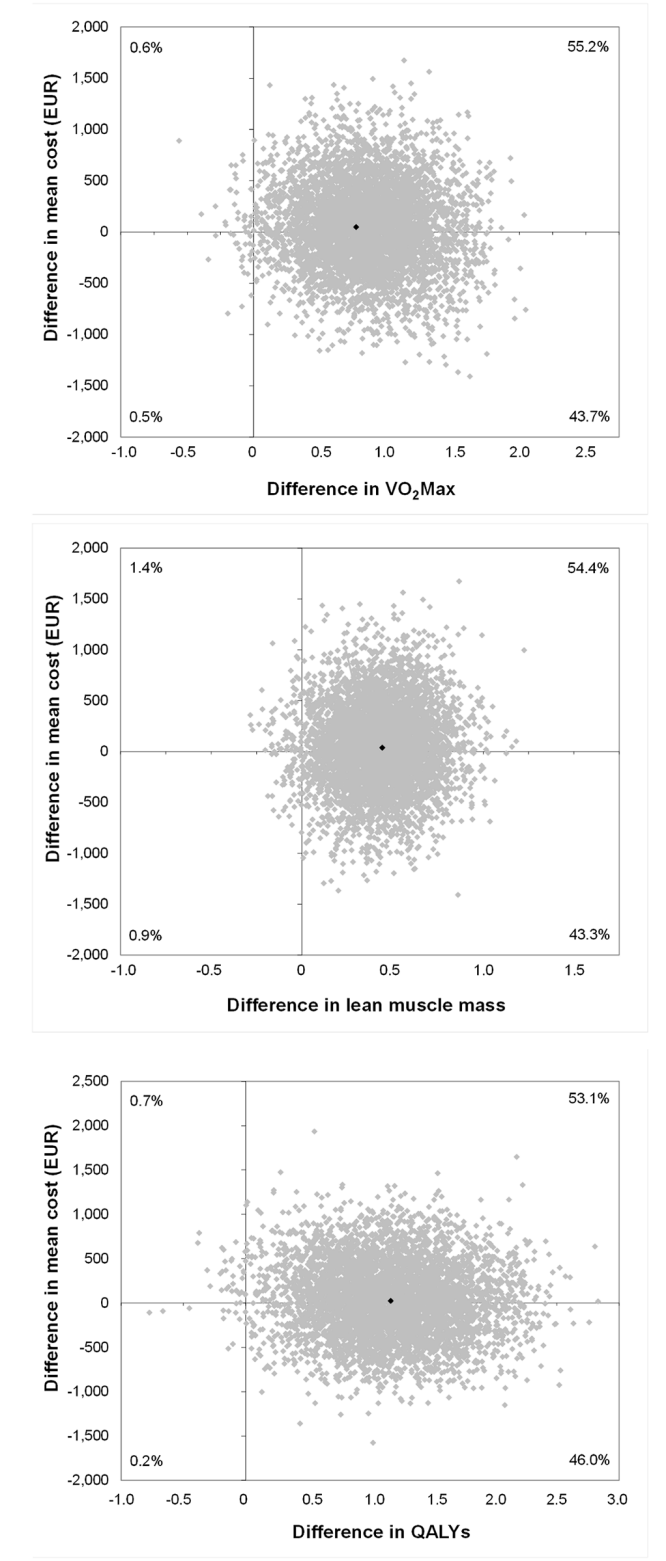
Cost-effectiveness plane for each QALY gained, cardiorespiratory fitness (ml/kg/min), and lean muscle mass.

**Fig 2:**
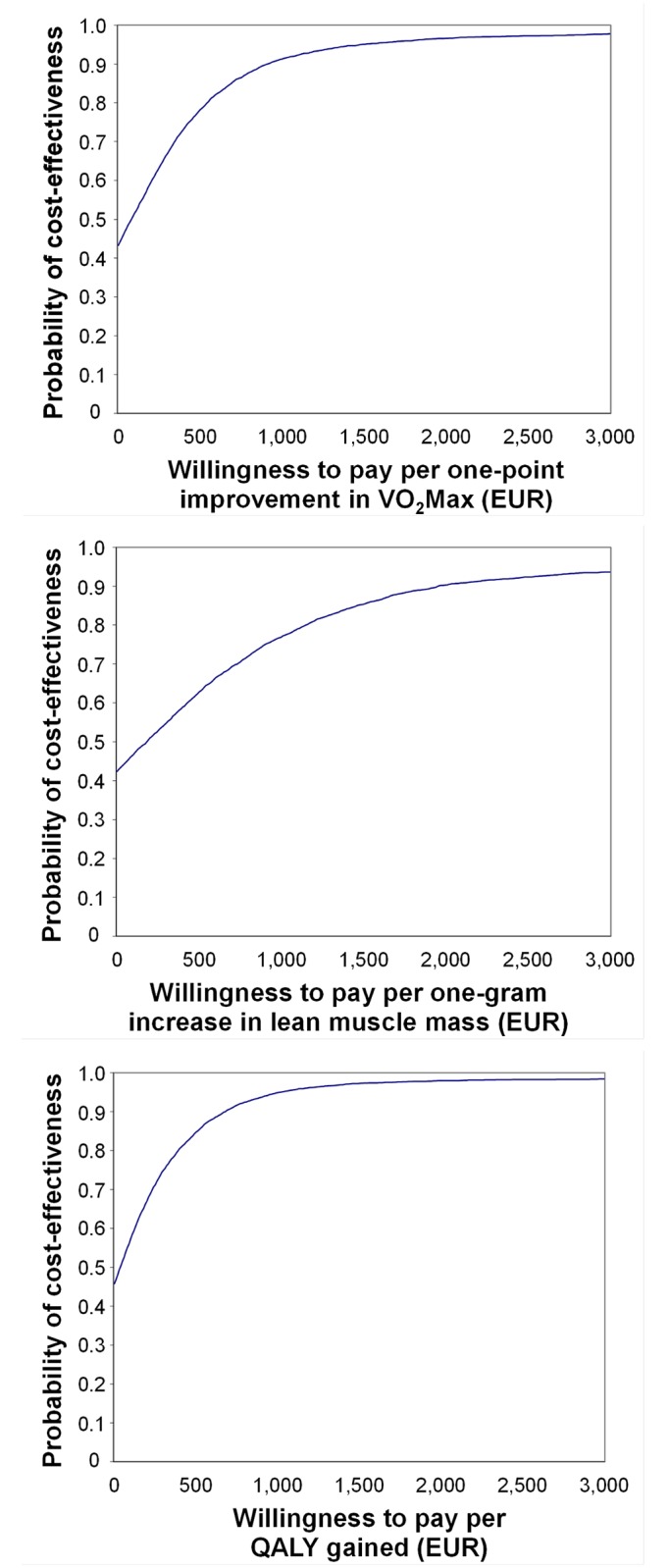
Acceptability curve for each QALY gained, cardiorespiratory fitness (ml/kg/min), and lean muscle mass.

The difference between cost values relative to maximal oxygen consumption was €54 (95% CI: -€740 to €914) and for effects relative to maximal oxygen consumption 0.86 ml/kg/min (95% CI: 0.15 to 1.60). The ICER was €63 for maximal oxygen consumption (see Table 3). As the cost-effectiveness plane indicates (see Fig 1), the intervention in VO_2_Max terms was more effective but there were no statistically significant differences in costs in comparison to the control group. Thus the data show that a one-unit improvement in cardiorespiratory fitness measured in ml/kg/min requires an additional cost of €63 per person. The second ICER was €126 per unit lean muscle mass, which represents a requirement for €126 additional cost for a one-gram increase in lean muscle mass in menopausal women. The final ICER was €46 per QALY gained (see Table 3). The cost-effectiveness acceptability curves indicate the probability of cost-effectiveness for various threshold values (see Fig 2). There is a 95% probability that the intervention would be cost-effective if society were willing to pay €1,398 per one-point improvement in VO_2_Max, €3,518 per one-gram increase in lean muscle mass, and €1,002 per QALY gained.

**Table 3:** Mean cost and effect differences (95% CI) between the intervention and control group in the main analysis and the sensitivity analysis, including incremental cost-effectiveness ratios and cost-effectiveness plane distributions.

	Sample size		Costs (EUR)	Effects	ICER	Distribution on CE plane (%)			
	Intervention group	Control group	Δ of intervention—usual care (95% CI)	Δ of intervention—usual care (95% CI)		NE	SE	SW	NW
**Main analysis**									
Maximal oxygen consumption (VO_2_Max)	74	77	54.0 (-740 to 914)	0.86 (0.15 to 1.60)	62.6	55.2	43.7	0.5	0.6
Lean muscle mass (kg)	74	77	54.0 (-740 to 914)	0.43 (0.01 to 0.83)	125.5	54.4	43.3	0.9	1.4
QALYs	73	77	53.0 (-710 to 930)	1.16 (0.21 to 2.11)	45.9	53.1	46.0	0.2	0.7
**Doubled aerobic training instructors’ costs**									
Maximal oxygen consumption	74	77	148 (-647 to 1008)	0.86 (0.15 to 1.60)	171.2	64.0	34.9	0.4	0.6
Lean muscle mass (kg)	74	77	148 (-647 to 1008)	0.43 (0.01 to 0.83)	343.0	63.1	34.6	0.8	1.5
QALYs	73	77	147 (-617 to 1023)	1.16 (0.21 to 2.11)	126.8	61.7	37.5	0.1	0.7
**Equal phone and heart-monitor costs**									
Maximal oxygen consumption	74	77	4.02 (-790 to 864)	0.86 (0.15 to 1.60)	4.7	50.1	48.9	0.5	0.5
Lean muscle mass (kg)	74	77	4.02 (-790 to 864)	0.43 (0.01 to 0.83)	9.3	49.5	48.3	1.2	1.2
QALYs	73	77	3.09 (-760 to 880)	1.16 (0.21 to 2.11)	2.7	48.3	50.8	0.3	0.6
**Complete case analysis**									
Maximal oxygen consumption	56	69	-91.2 (-912 to 813)	0.95 (0.22 to 1.66)	-95.8	41.4	58.0	0.3	0.3
Lean muscle mass (kg)	56	69	-91.2 (-912 to 813)	0.43 (-0.01 to 0.88)	-213.8	40.3	56.6	1.6	1.5
QALYs	56	69	-91.2 (-912 to 813)	1.22 (0.27 to 2.19)	-75.0	41.4	58.1	0.2	0.3

### Sensitivity analysis

The first sensitivity analysis applied the assumption that the cost of the aerobic training instructor’s work contribution would be two times higher per person, which means an increase in personal time from 3.5 to seven hours in total for the six months of the intervention. The results were quite similar to those in the main analysis, since additional positive effects of the physical-activity intervention were achieved at a low price (see Table 3). Neither the results with equal mobile-phone and heart-monitor costs between the two groups nor the case analysis carried out showed fundamental differences in comparison to the main analysis (see Table 3).

## Discussion

The intervention was cost-effective in terms of cardiorespiratory fitness, lean muscle mass, and QALYs gained: additional positive effects of physical activity were gained at a very low price [23]. The findings may point to considerable cost savings to be gained and positive effects on individuals’ health, functional ability, and work ability, if the higher level of physical activity is sustained after the intervention.

Menopause may predispose one to a decrease in muscle strength, which can have a negative effect on ability to work. However, both groups were of average cardiorespiratory fitness for their age and gender, which is enough for non-work tasks such as shovelling or performing heavy cleaning. According to Shephard [24], oxygen transport in a sedentary person’s body deteriorates by, on average, 5 ml/kg/min per decade and slightly more slowly in vigorously active persons. In the absence of physical activity, muscle strength decreases by 12–25% between ages 45 and 65, which may also reduce work ability and increase the likelihood of early retirement [10]. Moreover, lean muscle mass decreases because of ageing, and the lost muscle mass is replaced with fatty tissue [1]. As for functional ability, the decreased cardiorespiratory fitness, the lower muscle mass, and the higher quantity of fatty tissue may have negative effect on walking speed in addition to functional ability and work ability. From the perspective of society, if the positive effects of the intervention described in this paper remain, women who have physically demanding jobs may be assisted in maintaining their ability to work, which confers a huge societal advantage because every instance of an avoided disability pension saves the municipality costs of, on average, €60,000 in Finland [25].

While positive effects of physical activity on cardiorespiratory fitness were seen, increased fitness levels did not affect the number of days of illness. However, engaging in vigorous-level physical activity three times a week has been linked to a lower risk of sick leave; moderate physical activity, meanwhile, had no positive effect on number of days of illness [26]. In addition, according to a review by Amlani and Munir [27], regular endurance training may reduce sickness-related absence.

From the cost standpoint, the six-month duration of the intervention may have been too short to highlight effects of physical activity on the level of use of health-care services or the use of medication. We have published long-term follow-up results from the present cohort pertaining to work ability [28]. After 2.5 years, participants in the intervention group had higher work ability, as measured by means of the Work Ability Index, than at the beginning [28]. According to a previous study [29], physically active people use health-care services less than inactive people do. In addition, greater physical activity may help to keep further health-care costs and productivity losses under control by preventing symptoms related to menopause. Increased physical activity may also prevent chronic diseases, such as cardiovascular diseases, which cause, on average, nine per cent of health-care costs in Europe [30].

Although there are no cost-effectiveness studies related to physical-activity interventions for menopausal women, recent systematic reviews [31–33] have shown physical activity to be cost-effective for healthy adults in general. The effectiveness-related results of our study were similar to findings in previous trials, which showed that at least moderate-intensity walking is one way to gain health benefits, including improved cardiorespiratory fitness, and a decrease in fat tissue in menopausal women [7, 34]. In addition, it is noteworthy that menopause, with its many unpleasant symptoms (hot flushes, sleep disorders, night sweats, mood changes, etc.) is a favourable time at which to increase one’s physical activity in order to ease such symptoms and enjoy higher quality of life [8, 9, 35, 36]. At the same time, there are findings that contradict ours: some studies have shown no positive effect of regular physical activity on menopause symptoms, which may influence quality of life [37–40] or physical activity and might even increase hot flushes [35]. The connection between physical-activity status and symptoms of menopause is not simple; for example, obese women (BMI ≥ 30) report significantly more vasomotor and somatic symptoms than do normal-weight or overweight women [38]. Also, women with lower fitness levels report more hot flushes after moderate-intensity physical activity than physically active women do [41].

The use of mobile phones in the data collection was a strength of the study, even if the data received via the phones were not used in the cost-effectiveness analysis. In addition to reporting hot flushes, participants could contact the researchers, which may have been a motivating factor and increased adherence to the study. The objectively collected physical-activity-intensity data (from heart-rate monitor belts) constituted another strength of the study—according to Prince and colleagues [42], subjectively evaluated levels of physical activity as reported in questionnaires may involve both over- and under-reporting. However, recall bias may still have affected the results, with respect to the assessment of health-care utilisation and use of medication. The shortcomings with the control group involved the reliability of the subjectively collected physical-activity information and the feasibility of the instruction to keep one’s level of physical activity the same as before the intervention. Also, the study showed deficiencies to do with the opportunity cost of lost leisure time used for physical activity. In addition, the results may have been influenced by non-consideration of the travel expenses and time costs related to the use of health services. Preference weights obtained from a UK population may poorly characterise the results because Finnish people might differ from the UK population in their quality-of-life preferences. However, the questionnaire used for determination of quality of life has a greater effect. Sensitivity analyses showed that the main findings on cost-effectiveness were sustained even after inclusion of only complete cases—i.e., persons with non-missing data.

Symptoms of menopause are quite commonplace in the general ageing population, with 73% of postmenopausal women in Europe reporting hot flushes and almost half (45%) suffering from sleep disturbances [43]. Therefore, the findings were quite generalisable from this perspective. However, severely obese persons (BMI over 35) were excluded from the study population, which slightly restricts the generalisability of the findings because the worldwide prevalence of a BMI over 35 is 17.6% [44]. There is a need for more physical-activity trials aimed at evaluation of cost-effectiveness and for uncovering ways to achieve economic benefit via longer work careers, greater work efficiency, and prevention of early retirement and of chronic diseases alike, all toward the goal of increased HRQoL.

## Conclusions

During menopause, the risk of chronic diseases increases, and menopausal women are predisposed to a decrease in quality of life, in cardiorespiratory fitness, and in muscle strength and lean muscle mass. According to our findings, regular physical activity of at least moderate intensity among menopausal women was cost-effective for cardiorespiratory fitness, lean muscle mass, and gain in QALYs. The intervention may promote ability to work and thereby save on further costs of early retirement or disability pension. The length of follow-up may have been too short to reveal any change in use of health-care services or any productivity losses related to physical activity. More physical-activity trials are necessary for evaluation of cost-effectiveness on the road to finding ways to gain economic benefit by lengthening work careers, increasing work efficiency, and preventing early retirement.
